# Long-term outcomes of a paediatric quality improvement project in Central Asia: changes take time, time for a change

**DOI:** 10.7189/jogh.15.04133

**Published:** 2025-03-28

**Authors:** Sophie Jullien, Shamsov Bakhtovar Abdulkhafizovich, Rabiia Allakhveranova, Manzura Mirsaidova, Gulmira Nazhimidinova, Nurshaim Tilenbaeva, Shoira Yusupova, Martin W Weber, Susanne Carai

**Affiliations:** 1 Quality of care and patient safety office, World Health Organization, Athens, Greece; 2 Republican Scientific and Clinical Center of Pediatrics and Child Surgery, Ministry of Health of Tajikistan, Dushanbe, Tajikistan; 3 Research and Development Department, Central Asian International Consulting, Bishkek, Kyrgyz Republic; 4 International Higher School of Medicine, Bishkek, Kyrgyz Republic; 5 The Kyrgyz Republic Country Office, World Health Organization, Bishkek, Kyrgyz Republic; 6 Tajikistan Country Office, World Health Organization, Dushanbe, Tajikistan; 7 Witten/Herdecke University, Witten, Germany

## Abstract

**Background:**

Quality health care is essential for reducing child mortality. A three-year World Health Organization (WHO) quality improvement (QI) project, implemented in the Kyrgyz Republic and Tajikistan between 2012 and 2014, aimed to enhance the quality of paediatric hospital care and thereby reduce child mortality. The intervention included training on international guidelines, provision of medicines, supplies, and equipment, and supportive supervision. This study assessed whether the project was successful in improving clinical practices in the long term in both countries.

**Methods:**

We matched intervention hospitals with hospitals that did not participate in the QI project (control hospitals). We randomly selected medical records of children aged 2–59 months who were hospitalised with an acute respiratory infection or diarrhoea before the start of the QI project (2012), at its end (2015), and seven years after its completion (2021). We reviewed clinical practices from medical records to assess compliance with WHO standards for clinical care of children, which were emphasised in the project’s training sessions.

**Results:**

In the Kyrgyz Republic, the quality of care improved in intervention hospitals between the start and the end of the QI project for all indicators except one: unnecessary hospitalisations, unnecessarily prolonged hospitalisations, and unnecessary antibiotic prescriptions decreased, while the use of pulse oximetry and oral rehydration salts (ORS) prescriptions increased. This improvement was sustained until 2021. In control hospitals, some improvements were also observed between 2012 and 2015, but these were less substantial and less sustained. The interventions had less effect in Tajikistan between 2012 and 2015, and the improvements were not always sustained until 2021: unnecessary antibiotic prescriptions decreased and ORS prescriptions increased by 2015 but reverted to baseline levels by 2021.

**Conclusions:**

The QI project resulted in improvements in clinical practice in both countries, which were sustainable in the long term only in one country. The differences in long-term benefits may be attributable to factors within the health system environment. Issues related to health governance for, health financing, and health workforce were largely disregarded during the project’s design and implementation, yet may be crucial for sustainability.

The provision of good quality health care is key to reduce child deaths and accelerate progress towards achieving the health targets of the Sustainable Development Goals (SDGs) [1]. More than eight million people in low- and middle-income countries (LMICs) die every year from conditions that would have been treatable with high-quality health systems [2]. Of these, five million are people who accessed the health system but received poor-quality care [2]. For example, children receive less than half of the clinical recommended care, diagnoses are often incorrect, and safety concerns are frequent [2].

Growing evidence of the impact of poor quality has led to a stronger focus on adopting quality improvement strategies in LMICs. The World Health Organization (WHO) has invested in several such initiatives in different continents and developed guidance to improve the situation [3-5]. In the European Region, in 2012, WHO engaged with the Ministries of Health of the Kyrgyz Republic and Tajikistan in a three-year project aimed at strengthening their national health systems’ capacity to provide high-quality paediatric care and thus reducing child mortality [6]. In this project, a baseline assessment was conducted in 10 hospitals in each country, using a WHO quality assessment and improvement tool for hospital paediatric care [7]. It consisted of multiple indicators for the evaluation of hospital support services, case management of common and severe diseases, policies, and organisation of services. Case management was assessed by comparing practice with standards defined in the WHO pocket book of hospital care for children [8]. Findings were presented to key national stakeholders to develop an action plan that considered the identified weaknesses and key challenges.

A series of interventions followed for two years: training on international guidelines (WHO pocket book of hospital care for children) adapted to the country contexts; provision of medicines, supplies and equipment; and supportive supervision visits. These interventions were oriented to support the hospitals in achieving their action plan for improving quality of care, expand implementation to the national level, update national guidelines, and introduce paediatric care standards into national education and training for health professionals to ensure project sustainability. The endline assessment conducted upon completion of the project in 2014, using the same WHO assessment tool and compared to the baseline assessment, showed improved quality of paediatric care in most of the participating hospitals [6]. More specifically, an improved quality of care in managing children with severe diarrhoea and pneumonia, as well as a reduction in unnecessary hospitalisations, unjustified painful procedures, and unnecessary medicines [6,9].

What remains nearly a decade later? Scaling-up and sustainability are key in the achievement of high-quality health services and health goals [10]. As WHO is embarking on similar projects for improving quality of hospital care for mothers, newborns, and children, there is a need to assess the sustainability and long-term benefits of the former project as well as to identify areas with reversed improvements and persisting challenges. The aim of this study is to assess which improvements were still in place in the two countries a decade later. Because changes take time, long-term follow-up could also identify improvements that were not yet visible at the end of the project. Findings will offer pertinent insights into the selection and implementation of interventions for upcoming projects, to ensure sustainable improvements in the delivery of high-quality paediatric care.

## METHODS

### Study design

We conducted an interrupted time series analysis. Between September 2021 and March 2023, two research teams travelled to the hospitals that participated in the WHO 2012–2014 quality improvement (QI) project (hereafter referred to as intervention hospitals) and to hospitals not included in the project (hereafter referred to as control hospitals) for retrospective data collection from medical records. The researchers reviewed medical records of children hospitalised during three time periods: 2012 (prior to the start of the QI project), 2015 (at the end of the QI project), and 2021 (for assessment of long-term benefits of the QI project). None of the researchers in this study had participated in the implementation of the QI project.

### Hospital selection

The hospital and medical records selection process is summarised in Figure S1 in the **Online Supplementary Document**. In the Kyrgyz Republic, 10 hospitals were selected in 2012 to be part of the QI project and 10 hospitals with no intervention were identified in the context of a cluster randomised controlled trial [9]. Among those 20 hospitals, we collected data from five intervention hospitals and five control hospitals, which were purposely selected for geographical representation (Figure S2 in the **Online Supplementary Document**).

In Tajikistan, data were collected from eight out of the 10 hospitals that were part of the 2012–2014 QI project. No control hospitals had been previously identified. Therefore, we collected data from the 10 hospitals selected for the upcoming (at the time of study proposal) QI project as control hospitals, as these hospitals had no interventions prior to 2021 (Figure S3 in the **Online Supplementary Document**).

In both countries, the hospitals are district or regional public hospitals.

### Case record selection

In each hospital, we randomly selected medical records of children hospitalised in 2012, 2015, and 2021. We selected the medical records by picking one out of every three (or every ten in the biggest hospitals) from the piles of paper-based records, until obtaining the required number of records. We chose the number of 40 medical records to review based on previous similar work [9]. However, this number had to be reduced to 20 in some hospitals in Tajikistan due to logistical and time constraints. From the control hospitals in Tajikistan, data were collected from patients hospitalised in 2021 in the context of a health system evaluation [11]. Data from 2012 and 2015 were not collected in these hospitals initially and could not be collected later due to logistical constraints.

### Inclusion criteria

We reviewed medical records of children 2–59 months of age hospitalised with a primary diagnosis of an acute respiratory infection (upper respiratory infection, pneumonia, acute bronchitis, acute bronchiolitis or other acute lower respiratory tract infection) or diarrhoea (acute gastroenteritis), as they are the most common causes of paediatric hospitalisation in both countries [12].

### Indicators, standards of care, and determination of unnecessary and unnecessarily prolonged hospitalisations

**Figure 1:**
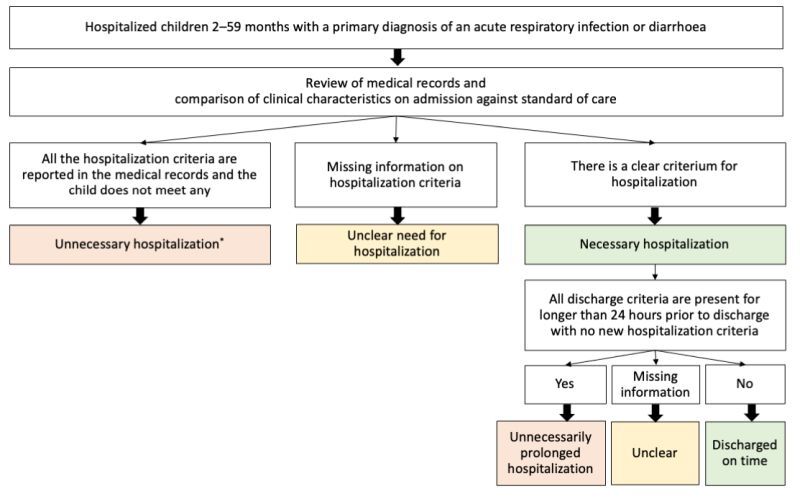
The Algorithm for classification of unnecessary and unnecessarily prolonged hospitalisations. *All unnecessary hospitalisations were considered as unnecessarily prolonged.

Indicators were selected based on the key findings reported in the baseline and endline assessments of the WHO QI project [6]: unnecessary hospitalisations, unnecessarily prolonged hospitalisations, use of pulse oximeter, and prescription of antimicrobials, oral rehydration salts (ORS), zinc, theophylline, and calcium gluconate. For the classification of each hospitalisation into necessary or unnecessary, the reference for standards of care was the WHO pocket book of hospital care for children as it has been adapted and adopted by the two countries as a reference manual for inpatient care, is broadly used in both countries and has previously been utilised in similar assessments [8,9]. To determine whether hospitalisation was necessary or not, we reviewed medical records for clinical characteristics present at the time of admission for the primary condition leading to hospitalisation and compared them against standard of care. Children with necessary hospitalisation were further classified as having unnecessarily prolonged hospitalisation if all discharge criteria were met for more than 24 hours before discharge, without any new hospitalisation criteria (**Figure 1**; Table S1 in the **Online Supplementary Document**).

### Data collection, management and analysis

We extracted data from medical records including general patient characteristics, hospitalisation and discharge dates, primary and secondary diagnoses, oxygen saturation measurement, and all medications received during hospitalisation. These data together with the classification of necessary or unnecessary hospitalisation and prolonged hospitalisation were directly entered into a digital database using predefined answers in prepopulated dropdown menus where applicable (Excel file). Prior to data collection, data collectors were trained by the same researcher for both countries to ensure consistency and comparability between hospitals and countries. We calculated cluster-level summary statistics by study arm (intervention and control hospitals), country, and year. We analysed data using Stata 18.0 (Stata Corporation LLC, College Station, USA) and used Microsoft Excel for graphs.

## RESULTS

Overall, 2095 medical records were reviewed and included in the analysis. Their general characteristics are summarised in **Table 1**.

**Table 1:** General characteristics of children hospitalised in intervention and control hospitals in Kyrgyzstan and Tajikistan, in 2012, 2015 and 2021

Variable	Characteristic	Type of hospital	Kyrgyzstan				Tajikistan			
			**All (n = 1182)**	**2012 (n = 390)**	**2015 (n = 397)**	**2021 (n = 395)**	**All (n = 913)**	**2012 (n = 158)**	**2015 (n = 181)**	**2021 (n = 574)**
			**IH: n = 593 CH: n = 589**	**IH: n = 196 CH: n = 194**	**IH: n = 200 CH: n = 197**	**IH: n = 197 CH: n = 198**	**IH: n = 561 CH: n = 352**	**IH: n = 158 CH: n = 0**	**IH: n = 181 CH: n = 0**	**IH: n = 222 CH: n = 352**
**Age (months)**	All children, median (Q1-Q3)	IH	13 (7–23)	10 (6–19)	14 (7–21)	15 (9–25)	11 (5–18)	9 (5–17)	10 (6–15)	11 (5–20)
		CH	14 (7–25)	13 (7–24)	13 (7–22)	16.5 (8–30)	12 (7–20)	NA	NA	12 (7–20)
	Infants (2–11 mo)	IH	45.0%	54.6%	43.5%	37.1%	56.5%	58.9%	58.0%	53.6%
		CH	41.1%	44.9%	44.2%	34.3%	46.9%	NA	NA	46.9%
**Referral from PHC or other hospitals**		IH	60.9%	63.8%	68.5%	50.3%	45.3%	46.2%	49.2%	41.4%
		CH	65.2%	69.1%	72.1%	54.6%	53.1%	NA	NA	53.1%
**Primary diagnosis at admission**	Acute respiratory infection	IH	8.4%	9.7%	7.5%	8.1%	39.6%	34.8%	35.4%	46.4%
		CH	9.2%	13.4%	3.6%	10.6%	31.5%	NA	NA	31.5%
	Pneumonia	IH	31.0%	17.4%	34.5%	41.1%	21.2%	24.7%	24.9%	15.8%
		CH	30.1%	30.9%	33.0%	26.3%	16.5%	NA	NA	16.5%
	Acute bronchitis/bronchiolitis	IH	17.2%	21.4%	18.0%	12.2%	11.9%	21.5%	6.1%	9.9%
		CH	22.4%	26.3%	15.7%	25.3%	18.2%	NA	NA	18.2%
	Acute gastroenteritis	IH	43.3%	51.5%	40.0%	38.6%	27.3%	19.0%	33.7%	27.9%
		CH	38.4%	29.4%	47.7%	37.9%	33.8%	NA	NA	33.8%
**Other diagnoses (most common)**	Anaemia	IH	48.2%	51.0%	51.0%	42.6%	36.2%	38.6%	35.4%	35.1%
		CH	38.0%	41.8%	45.7%	26.8%	47.2%	NA	NA	47.2%
	Seizure	IH	2.0%	1.5%	3.0%	1.5%	7.8%	7.0%	9.4%	7.2%
		CH	3.9%	3.1%	3.1%	5.6%	1.1%	NA	NA	1.1%
	Urinary tract infection	IH	2.2%	1.5%	3.5%	1.5%	0.0%	0.0%	0.0%	0.0%
		CH	1.5%	1.6%	1.5%	1.5%	0.0%	NA	NA	0.0%
	Acute tonsillitis	IH	0.8%	0.0%	1.0%	1.5%	0.0%	0.0%	0.0%	0.0%
		CH	0.9%	1.0%	0.5%	1.0%	2.0%	NA	NA	2.0%
	Neurotoxicosis*	IH	0.7%	1.0%	0.5%	0.5%	11.4%	10.8%	5.0%	17.1%
		CH	0.7%	1.0%	1.0%	0.0%	13.6%	NA	NA	13.6%
	Perinatal encephalopathy	IH	0.5%	0.5%	1.0%	0.0%	3.0%	3.2%	3.9%	2.3%
		CH	0.7%	1.6%	0.5%	0.0%	6.0%	NA	NA	6.0%
	Malnutrition	IH	0.7%	0.0%	2.0%	0.0%	2.9%	5.1%	2.8%	1.4%
		CH	0.3%	0.5%	0.5%	0.0%	10.2%	NA	NA	10.2%
**Hospitalisation in ICU**		IH	4.2%	4.6%	6.0%	2.0%	7.3%	7.0%	5.0%	9.5%
		CH	6.1%	4.6%	9.6%	4.0%	2.3%	NA	NA	2.3%

CH – control hospitals, ICU – intensive care unit, IH – intervention hospitals, NA – no answer, PHC – primary health care, Q – quartile

*Neurotoxicosis is not a diagnosis included in ICD-10 but was used in the medical records as an umbrella term to refer to neurological dysfunction as a complication of an infectious disease.

In the Kyrgyz Republic, infants (2–11 months) accounted for 45.0 and 41.1% of the children across the intervention and control hospitals, respectively. In Tajikistan, 56.5 and 46.9% were infants across the intervention and control hospitals, respectively. In the Kyrgyz Republic, 60.9% of children in the intervention hospitals and 62.5% in the control hospitals were referred from primary healthcare or other hospitals. In Tajikistan, this was the case for 45.3 and 53.1% of children, respectively.

In the Kyrgyz Republic, among the selected medical records, the most common primary diagnosis was diarrhoea, followed by pneumonia, acute bronchitis and bronchiolitis, and acute respiratory infections, as recorded by health care providers. In Tajikistan, the most common primary diagnosis at admission was acute respiratory infection, followed by diarrhoea, pneumonia, and acute bronchitis and acute bronchiolitis. Among other diagnoses documented during hospitalisation, anaemia was by far the most common in both countries, followed by ‘neurotoxicosis’ and malnutrition in Tajikistan.

The main findings are summarised in Table S2 in the **Online Supplementary Document**.

### Unnecessary and unnecessarily prolonged hospitalisations

Standards of care: Children should be hospitalised only when and for the time that is strictly required [13].

**Figure 2:**
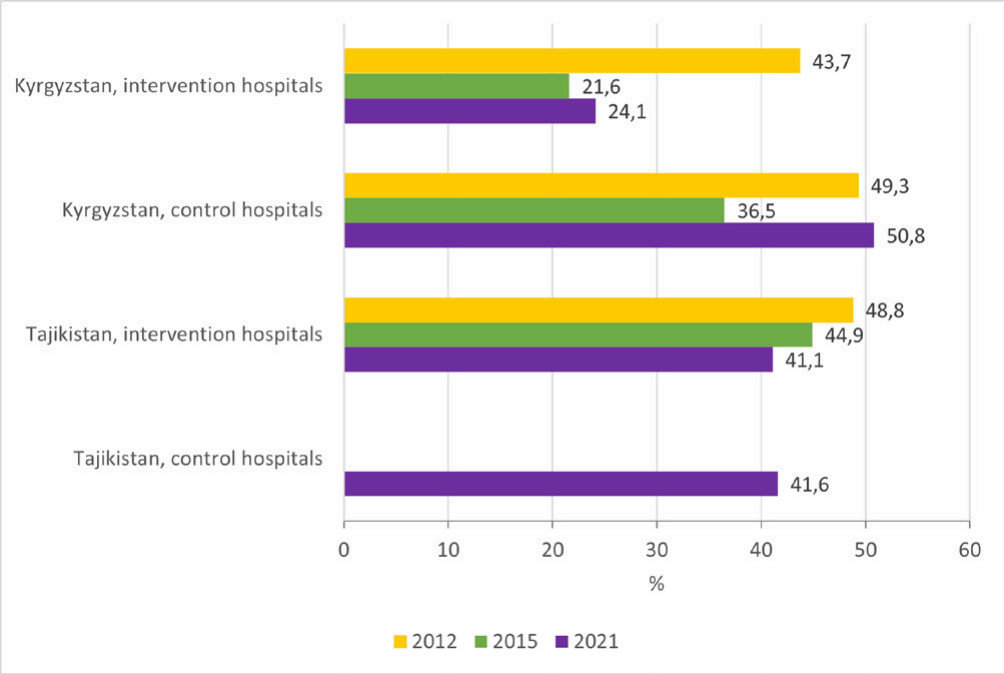
Proportion of unnecessary hospitalisations.

Findings: The proportion of unnecessary hospitalisations in the Kyrgyz Republic decreased from 43.7 to 21.6% in the intervention hospitals between the start and the end of the QI project, with sustained improvements in 2021 (24.1%) (**Figure 2**; Table S3 in the **Online Supplementary Document**). In Tajikistan, the proportion of unnecessary hospitalisations slightly decreased from 48.8 to 44.9% over the QI project in intervention hospitals, remaining at 41.1% in 2021, similar to the situation in control hospitals (41.6% in 2021) (**Figure 2**; Table S4–5 in the **Online Supplementary Document**).

**Figure 3:**
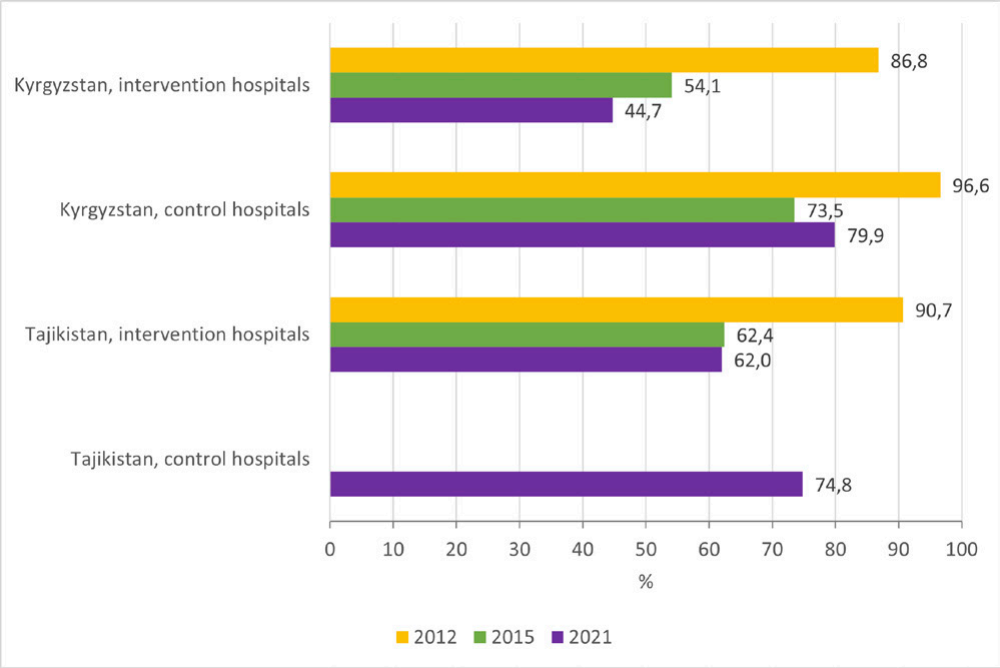
Proportion of unnecessarily prolonged hospitalisations.

Unnecessarily prolonged hospitalisations decreased in both countries in intervention hospitals during the QI project, with sustainable improvements in 2021, which was not the case in control hospitals (**Figure 3**; Table S2–5 in the **Online Supplementary Document**).

### Use of pulse oximeter

Standards of care: Oxygen saturation must be checked with a pulse oximeter and recorded upon admission of all children with a respiratory condition, to guide the possible use of oxygen [8].

**Figure 4:**
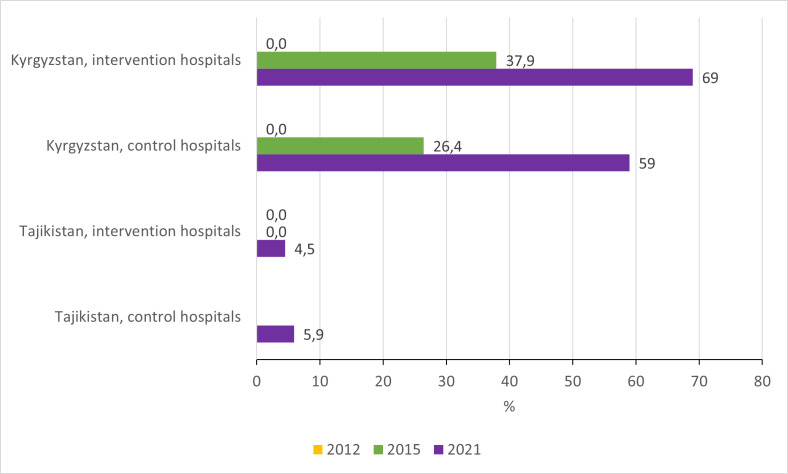
Proportion of children with a primary diagnosis of a respiratory infection for which pulse oximetry measurement was recorded at least once upon admission and during hospitalisation.

Findings: The use of pulse oximeter among children with a primary diagnosis of a respiratory condition at admission increased in all the hospitals in the Kyrgyz Republic, while its use in 2021 remains very low in Tajikistan (**Figure 4**; Table S2–5 in the **Online Supplementary Document**).

### Antimicrobials prescription during hospitalisation

Standards of care: Antibiotic prescription is justified in children with pneumonia, but antibiotics are not needed for all children with acute bronchitis, acute bronchiolitis and acute respiratory infections [8]. Children with diarrhoea (dysentery excluded) do not need antibiotics [8].

**Figure 5:**
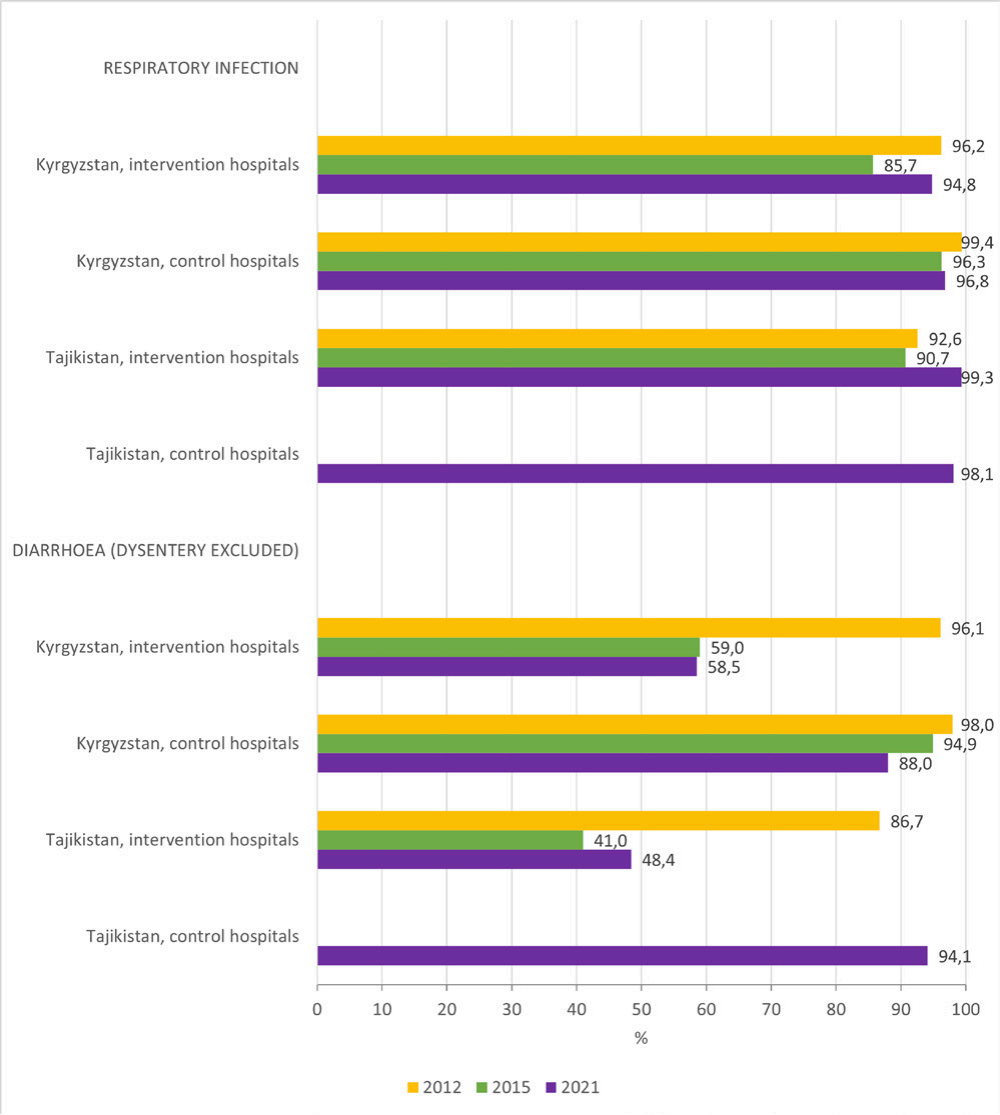
Proportion of children who were prescribed antibiotics, disaggregated by primary diagnosis.

Findings: Most children hospitalised with a respiratory infection received at least one antimicrobial during hospitalisation (**Figure 5**; Table S2–5 in the **Online Supplementary Document**). In 2012, around 95% of children with diarrhoea (dysentery excluded) were prescribed antibiotics. By the end of the QI project, the proportion of unjustified antibiotic prescriptions in children with diarrhoea reduced in intervention hospitals to 59.0% (Kyrgyz Republic) and 41.0% (Tajikistan). Improved results were sustained in 2021 in both countries while unjustified prescription remained high (over 85%) in control hospitals (**Figure 5**; Table S2–5 in the **Online Supplementary Document**).

### Oral rehydration salts (ORS) and zinc prescription in children with diarrhoea

Standards of care: Children hospitalised with diarrhoea should receive ORS and zinc [8].

**Figure 6:**
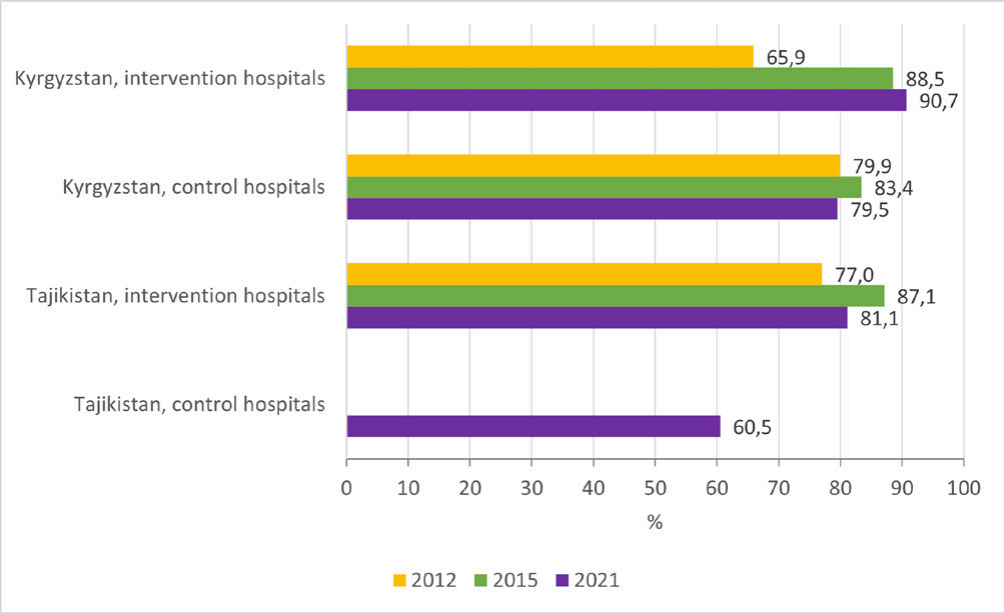
Proportion of children hospitalised with a primary diagnosis of diarrhoea who were prescribed oral rehydration salts.

Findings: The proportion of children with diarrhoea who were prescribed ORS increased between 2012 and 2015 in intervention hospitals in both countries but was only sustained in the Kyrgyz Republic. However, the proportion of ORS prescription in 2021 was higher in intervention hospitals than in control hospitals in the two countries (**Figure 6**; Table S2–5 in the **Online Supplementary Document**).

**Figure 7:**
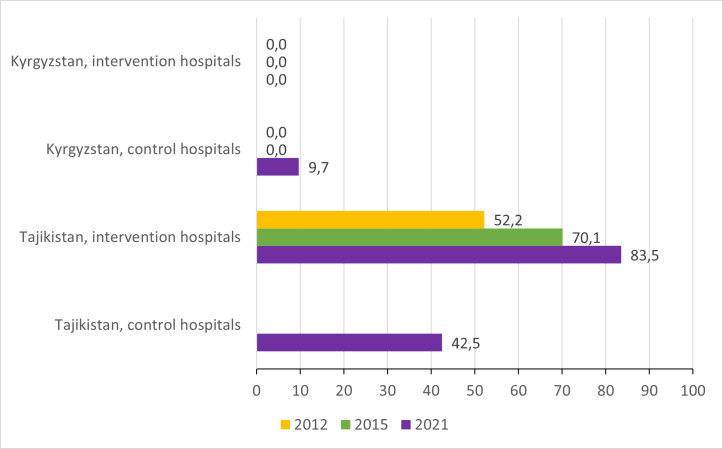
Proportion of children hospitalised with a primary diagnosis of diarrhoea who were prescribed zinc.

Prescription of zinc among children with diarrhoea increased considerably among intervention hospitals in Tajikistan, from 52,2% in 2012 to 83.5% in 2021, while zinc prescription remains almost nil in all the hospitals in the Kyrgyz Republic (**Figure 7**; Table S2–5 in the **Online Supplementary Document**).

### Theophylline and calcium gluconate prescription in children with a respiratory condition

Standards of care: Medications should be prescribed only when indicated. In both countries, the baseline assessment showed that children were commonly prescribed medications when they were not indicated or with no evidence of benefits, such as intravenous (IV) theophylline or calcium gluconate in children with a respiratory infection [14].

**Figure 8:**
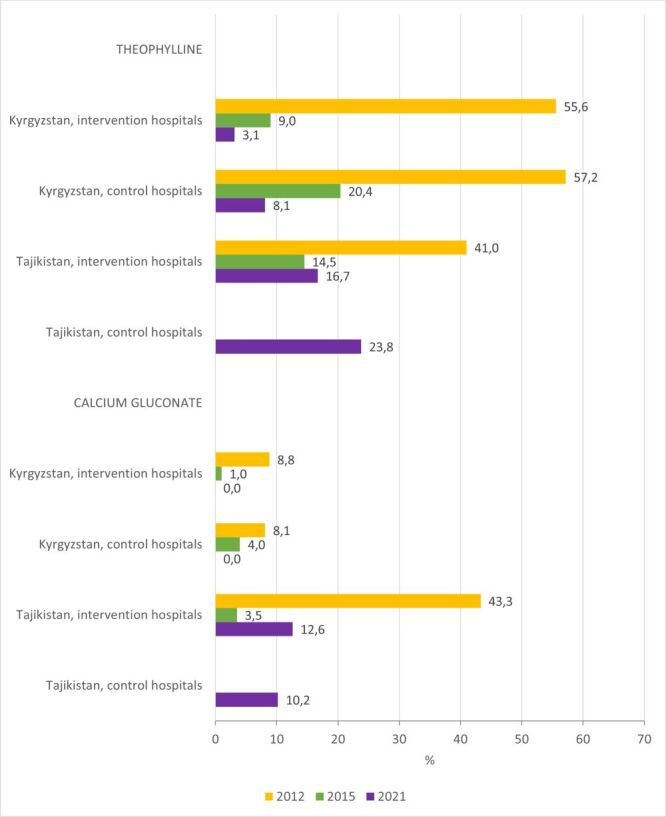
Proportion of children hospitalised with a primary diagnosis of a respiratory infection who were prescribed iv theophylline and calcium gluconate.

Findings: This practice improved in both countries by the end of the QI project, both in intervention and control hospitals (**Figure 8**; Table S2–5 in the **Online Supplementary Document**).

## DISCUSSION

### Sustained improvement in paediatric care in the Kyrgyz Republic

Findings from the Kyrgyz Republic show that focused training, supportive supervision, and provision of medicines, supplies, and equipment over a two-year period improved the quality of paediatric care in hospitals participating in the QI project, based on selected indicators. This improvement was sustained until 2021, seven years after the project ended. In the control hospitals in the Kyrgyz Republic, an improvement was also observed between 2012 and 2015. However, this was not sustained until 2021 for some of the indicators, including unnecessary hospitalisations and unnecessarily prolonged hospitalisations. The improvement in the control hospitals could be attributed to the Hawthorne effect, where doctors performed better knowing they were part of a study. It may also be due to a ripple effect, possibly caused by the partial replication of quality improvement approaches by other organisations in other hospitals, or by national supportive supervisors incorporating these methods into their on-the-job training curricula. Alternatively, it could be due to a trend that had reasons beyond the project, in which case however the regression to old practices after the end of the project could not be explained [15].

### Factors affecting quality of care

The inadequate quality of care arises from multiple factors, including limited health worker knowledge, lack of motivation and support systems, shortages of essential medicines, vaccines, and equipment, as well as insufficient financing and leadership [2]. These factors directly impeded the project's broader impact, as many improvements relied on robust infrastructure and consistent resource availability. For example, the provision of zinc and antibiotics aligned with international guidelines depends on supply chains, their prescription relies on health worker knowledge and context acceptance, and patient intake depends on the population’s trust in the health system as well as the affordability and accessibility of health care facilities.

Evidence from large systematic reviews suggests that multifaceted interventions, which include infrastructure strengthening, training, supportive supervision, and various management techniques, can effectively improve health care providers' practices [16-18]. Determining whether effects diminish over time is essential to improving sustainability. However, little is known about the effectiveness in the long term. A systematic review of 37 studies conducted in LMICs found that effects varied according to the type of interventions chosen to improve health care providers’ practices [19]. The median follow-up time was six months, which limits the ability to extrapolate these findings to longer term benefits. For training alone, effects tended to decline over time, while effects were maintained for group problem solving plus training and increased when the intervention consisted of group problem-solving alone. Group problem-solving strategies involve continuous quality improvement through ongoing cycles of planning (Plan), implementing (Do), monitoring (Check), and revising (Act) the strategy as it is executed. This approach, widely recognised as the PDCA or quality cycle, was the strategy employed in the QI project in Tajikistan and the Kyrgyz Republic [20].

### Contextual variations in LMICs

Other research has pointed out that the effectiveness of quality improvement interventions varies considerably in LMICs depending on the context [21,22]. In Tajikistan, the same interventions had less effect in improving quality of hospital care than in its neighbouring country. The quality of care improved in the hospitals that participated in the QI project for some indicators such as unnecessary hospitalisation (minor improvement), unnecessarily prolonged hospitalisation, antibiotic prescription in children with diarrhoea, and zinc prescription in children with diarrhoea. However, improvements were not sustained until 2021 for some of the indicators and there was no improvement for other indicators (*e.g*. use of pulse oximeter in children with a respiratory condition). No control hospitals were studied in Tajikistan before (2012) and at the end (2015) of the QI project. Data collected in 2021 from hospitals not previously involved in the QI project show performance trends that do not consistently align with those of the intervention hospitals.

### Persistent challenges in Central Asia

The indicators were selected based on the key findings from the baseline and endline assessments conducted by the WHO and national teams in 2012 and 2014 [6]. However, these issues of unnecessary and lengthy hospital stays as well as excessive and ineffective treatment including unjustified antibiotics are not new to the region. A similar assessment of the quality of hospital care for children conducted in Kazakhstan, the Republic of Moldova and the Russian Federation in 2002 already highlighted these critical areas [5]. Despite the efforts dedicated to improving these issues, these are persistent challenges in Central Asia [23].

Findings showed sustainable improvements linked to the QI project in the Kyrgyz Republic and Tajikistan for some indicators. However, in 2021, intervention hospitals continued to have between one in four and one in two children with unnecessary hospitalisations, about half of the children with unnecessarily prolonged hospitalisations, and over half of the children with diarrhoea receiving unjustified antibiotics; the situation was worse in control hospitals. If improved practices were truly embedded in the health system, further improvements and outcomes at national scale, including the control hospitals, would be expected in the long term. Why are sustainable and better outcomes not achieved for these persistent challenges, despite several projects aimed at addressing them?

### System-wide actions for high-quality health systems

Health care is delivered through health systems, which are complex adaptive systems functioning across multiple interconnected levels [2]. This study highlights that sustainable quality improvement requires system-wide actions to address core structural issues. Micro-level improvement interventions, such as training of health care providers or quality improvement at facility level, tend to struggle to improve the underlying performance of the whole system. Achieving high-quality health systems requires structural actions at all levels of the system [2]. First, sound governance with a quality-of-care vision is needed, supported by updated and enforced laws and regulations including for the private sector. Second, adequate investment in the health system is key, both in absolute and relative terms to GDP and government budget, with low out-of-pocket spending. Third, defining service delivery between hospitals and primary health care with clear referral pathways is crucial for achieving optimal health outcomes. Fourth, ensuring a well-trained, well-remunerated and well-respected workforce. Fifth, the population needs to be engaged, health-educated, and empowered for a responsible use of the health system, seeking evidence-based high-quality of care.

Therefore, unless development projects include actions at all these levels or are embedded within a network of partners so that all levels are addressed, improving quality of care is unlikely to be efficient and sustainable. For example, if health professionals are trained on the management of children with diarrhoea, we could expect a decrease in the unjustified prescription of antibiotics for these children. However, if the health professionals in the private sector continue to prescribe antibiotics, if pharmaceutical companies incentivise antibiotic prescription, if there are no regulations preventing the over-the-counter sale of antibiotics, and if the population perceives antibiotic prescription as good practice, then training health professionals on the appropriate indications for antibiotics as a single intervention is unlikely to result in improved quality care.

In this way, among the indicators that we selected for this study, a sustainable decrease in unnecessary hospitalisations and unnecessarily prolonged hospitalisations at the national level would require, at least, transformation of the health financing, strengthening of primary health care, and increased trust from the population in seeking care in PHC. In addition, enforcement of legislation around selling antibiotics and other medicines would be needed to decrease polypharmacy practices and antibiotics overuse. Zinc prescription is another clear example of the need for system-wide actions. In the Kyrgyz Republic, zinc is included in the national list of essential medicines and is produced by a pharmaceutical company in the capital since 2016. However, it is not available in most hospitals and pharmacies in the country. In Tajikistan, zinc is free of charge for children hospitalised with diarrhoea. According to the Ministry of Health of the Republic of Tajikistan, zinc is available in the pharmacies throughout the country since 2015 and has been distributed in hospitals and primary health care centres since before the start of the QI project, with no interruption in its administration (personal communication). Increasing the use of pulse oximeters and ORS prescriptions for children with diarrhoea, however, likely depends on improving health professionals' practices, provided these tools are available in health facilities and their use is acceptable to the population.

### Designing development projects

While designing development projects aimed at improving quality of care, all stakeholders must be fully aware of the complexity of the health system and understand how it performs. The health system performance assessment (HSPA) framework for universal health coverage illustrates the already aforementioned relevant health system areas, the so-called functions (governance, financing, resource generation and service delivery), and the influence they have on each other as well as to intermediate objectives (effectiveness, safety, user experience, access, efficiency and equity of service delivery) and final goals (people-centredness, health improvement, financial protection, efficiency and equity of the health system) [24]. High quality (intermediate objectives) is required for achieving the final goals of the health system but achieving high quality depends on the functionality of all the interlinked functions.

### Shift towards system-based solutions

For a long time, most efforts have been focused on the intermediate objectives for improving the final goals. It is time to shift from small scale (micro-level) quality improvement interventions towards systems-based solutions.

Changes take time. The challenge is that changes in health systems are often driven by independent development projects that are based on time-bound activities. These projects prioritise short-term outcomes that must be reported upon the completion of the (too short) projects to satisfy the donors. Longer timeframes in funding projects are surely needed for implementing effective and lasting results. In addition, consistent leadership with a long-term vision and commitment is key in the coordination and implementation of the different activities, under an overall strategic plan [25]. Frequent turnover in leadership is likely to hamper continuity in the support required for this process [26]. Finally, better coordination and transparency between international partners and the multiple actors are urgently needed to allow for the shift from independent micro-level interventions towards a comprehensive package of interventions at all levels.

### Limitations and strengths of the study

The main limitation of this research is that it is based on quantitative data collected retrospectively from medical reports. As such, the findings rely on the veracity of the information recorded by health workers in those documents. There is a strong punitive culture both in the Kyrgyz Republic and Tajikistan, reflected in the medical practice by regular control visits in the hospitals with review of the medical records. Such practices are likely to lead, in some cases, to information in the medical records that do not reflect the reality, as health workers may record data to meet the criteria known to be controlled for avoiding punishment. This could result in an overestimation of the improvements linked to the QI project.

A major strength of this study is the evaluation of outcomes seven years after the completion of the project, providing important long-term data. This significantly exceeds the longest follow-up period of 34 months reported in the 37 studies included in Arsenault's systematic review [27]. The use of the same methods and data collection tools for both countries is another major strength of this study, allowing analysis of trends and comparison between countries. The endline assessment of the QI project was conducted in 2014 with rigorous methodology, but by the same team involved in the implementation of the interventions, leading to potential detection and reporting bias. In this study, data collection, analysis and interpretation were performed by an independent team.

## CONCLUSIONS

There is no quality of care without a functioning health system. Sustainable improvements in health care quality hinge on addressing foundational issues across governance, financing, resources and trust. This requires coordinated changes at all levels of the health system. But changes take time. Thus, it is time to change. The key message is clear: achieving high-quality health care systems demands a shift in focus from short-term outcomes to integrated, system-wide changes. Funding agencies and international partners must align their actions to achieve what truly matters: sustainable improvements towards high-quality health care systems.

## Additional material


Online Supplementary Document
