# Supplementary material for: Long-term outcomes of a paediatric quality improvement project in Central Asia: changes take time, time for a change
Source: J Glob Health. 2025 Mar 28;15:04133. doi: 10.7189/jogh.15.04133 (PMC11950901; doi:10.7189/jogh.15.04133)

Supplement to: Jullien S, Abdulkhafizovich SB, Allakhveranova R, Mirsaidova M, Nazhimidinova G, Tilenbaeva N, Yusupova S, Weber MW, Carai S. Long-term outcomes of a paediatric quality improvement project in Central Asia: changes take time, time for a change. J Glob Health. 2025;15:04133.

Table S1. Standards of care for assessment of unnecessary and unnecessarily prolonged hospitalizations

Table S2. Summary of findings at baseline (2012), 2015, and 2021 in intervention and control hospitals in Kyrgyzstan and Tajikistan

Table S3: Findings at baseline (2012), 2015, and 2021 in intervention and control hospitals in Kyrgyzstan

Table S4: Findings at baseline (2012), 2015 and 2021 in intervention hospitals in Tajikistan

Table S5: Findings in control hospitals in Tajikistan (2021)

Fig S1. Hospital selection and medical records selection in Kyrgyzstan and Tajikistan

Fig S2. Intervention and control hospitals in Kyrgyzstan

Fig S3. Intervention and control hospitals in Tajikistan

Table S1. Standards of care for assessment of unnecessary and unnecessarily prolonged hospitalizations

Disease, condition	Hospitalization criteria[1]	Discharge criteria[1]
**FOR ALL**	The presentation of any **general danger sign** entails a hospitalization criterion by itself:<br>• inability to drink or breastfeed,<br>• vomiting everything,<br>• history of convulsions during the current illness,<br>• lethargy, unconsciousness or convulsions.	A decision on when to discharge should be made on an individual basis, taking into consideration factors such as:<br>• the family's home circumstances and how much support is available to care for the child<br>• the staff's judgement of the likelihood that the treatment course will be completed at home or that the family will return immediately to hospital if the child's condition worsens.
**Upper respiratory tract infection including common cold and croup**	Severe pneumonia (see "pneumonia" below)<br>Severe croup, defined by any of the following:<br>• Stridor even when the child is at rest<br>• Rapid breathing and low chest indrawing<br>• Oxygen saturation <90% or central cyanosis	• Respiratory distress resolved<br>• No hypoxaemia (SpO2>90%) on room air<br>• No apnoea<br>• No stridor<br>• Alert<br>• Afebrile<br>• No severe dehydration<br>• Feeding/eating well<br>• Not vomiting everything<br>• Able to take oral medication (if needed)
**Pneumonia**	Severe pneumonia, defined by any of the following:<br>• Oxygen saturation <90% or central cyanosis<br>• Severe respiratory distress (grunting, very severe chest indrawing)<br>Pneumonia not improving after three days (of oral antibiotics)	
**Acute bronchitis<br>Acute bronchiolitis<br>Unspecified acute lower respiratory tract infection**	• Oxygen saturation <90% or central cyanosis<br>• Apnoea or history of apnoea<br>• Gasping and grunting (especially in young infants)<br>• Not improving 15 minutes after administration of rapid-acting bronchodilator (e.g. salbutamol)<br>- Signs of severe pneumonia (see above)<br>- Fast breathing: ≥ 50 breaths/minute in 2–11 months, ≥ 40 breaths/minute in 1–5 years	
**Diarrhoea, acute gastroenteritis, intestinal infectious diseases**	Severe dehydration, defined as ≥ 2 of the following signs:<br>• lethargy or unconsciousness<br>• sunken eyes<br>• unable to drink or drinks poorly<br>• skin pinch goes back very slowly (≥ 2 sec) or "reduced turgor"	• No severe dehydration<br>• Feeding/eating well<br>• Not vomiting everything<br>• Alert<br>• Afebrile
	Severe persistent diarrhoea:<br>• diarrhoea lasting ≥ 14 days<br>• with signs of dehydration: see severe dehydration signs above, or ≥ 2 of the following signs: restlessness, irritability; sunken eyes; drinks eagerly, thirsty; skin pinch goes back slowly.	
	Dysentery (frequent loose stools mixed with blood) if any of the following criteria:<br>• < 2 months old<br>• severely ill children, who look lethargic, have abdominal distension and tenderness or convulsions	

[1] Based on the WHO *pocket book of Hospital care for children* [21].

Table S2: Summary of findings at baseline (2012), 2015, and 2021 in intervention and control hospitals in Kyrgyzstan and Tajikistan

		Intervention hospitals			Control hospitals		
		2012	***2015***	***2021***	***2012***	***2015***	***2021***
**Unnecessary hospitalizations (%)**	Kyrgyzstan	43.7 (22.6-87.1)	21.6 (0.0-57.6)	24.1 (0.0-61.5)	49.3 (5.5-93.1)	36.5 (0.0-78.7)	50.8 (6.9-94.6)
	Tajikistan	48.8 (14.2-83.5)	44.9 (10.4-79.4)	41.1 (7.0-75.2)	–	–	41.6 (11.1-72.2)
**Unnecessarily prolonged hospitalizations**	Kyrgyzstan	86.8 (57.2-100)	54.1 (10.4-97.8)	44.7(1.1-88.2)	95.6 (77.7-100)	73.5 (34.8-100)	79.9 (44.8-100)
	Tajikistan	90.7 (70.6-100)	62.4 (28.8-95.9)	62.0 (28.4-95.7)	–	–	74.8 (47.9-100)
**Use of pulse oximeter**	Kyrgyzstan	0.0	37.9 (0.0-80.4)	69.0(28.4-100)	0.0	26.4 (0.0-65.0)	59.0 (15.9-100)
	Tajikistan	0.0	0.0	4.5 (0.0-18.7)	–	–	5.9 (0.0-20.5)
**Antimicrobial prescription during hospitalization for diarrhoea**	Kyrgyzstan	96.1 (79.2-100)	59.0 (10.8-100)	58.5 (15.3-100)	98.0 (82.3-100)	94.9 (75.6-100)	88.0 (59.5-100)
	Tajikistan	86.7 (22.1-100)	41.0 (4.5-71.9)	48.4 (27.1-98.7)	–	–	94.1 (79.6-100)
**Antimicrobial prescription during hospitalization for respiratory infection**	Kyrgyzstan	96.2 (79.5-100)	85.7 (54.9-100)	94.8 (75.3-100)	99.4 (92.5-100)	96.3 (79.7-100)	96.8 (81.3-100)
	Tajikistan	92.6 (74.5-100)	90.7 (70.6-100)	99.3 (93.3-100)	–	–	98.1 (89.7-100)
**Oral rehydrated salts prescription[1]**	Kyrgyzstan	65.9 (24.3-100)	88.5 (57.2-100)	90.7 (65.2-100)	79.9 (34.6-100)	83.4 (50.9-100)	79.5 (44.2-100)
	Tajikistan	77.0 (40.0-100)	87.1 (36.8-100)	81.1 (52.1-100)	–	–	60.5(53.8-67.2)
**Zinc prescription[1]**	Kyrgyzstan	0.0	0.0	0.0	0.0	0.0	9.7(0.0-35.7)
	Tajikistan	52.2 (8.4-96.0)	70.1(38.4-100)	83.5 (56.1-100)	–	–	42.5 (11.9-73.1)
**Theophylline prescription[2]**	Kyrgyzstan	55.6 (12.1-99.2)	9.0 (0.0-34.0)	3.1 (0.0-18.2)	57.2 (13.8-100)	20.4 (0.0-55.7)	8.1(0.0-32.0)
	Tajikistan	41.0 (6.9-75.0)	14.5 (0.0-38.8)	16.7 (0.0-42.6)	–	–	23.8 (0.0 – 50.2)
**Calcium gluconate prescription[2]**	Kyrgyzstan	8.8 (0.0-33.7)	1.0 (0.0-9.7)	0.0	8.1(0.0-32.0)	4.0 (0.0-21.1)	0.0
	Tajikistan	43.3 (8.9-77.6)	3.5 (0.0-16.1)	12.6 (0.0-35.5)	–	–	10.2 (0.0 – 29.0)

95% CI: 95% Confidence Intervals; 1. Children with diarrhoea; 2. Children with respiratory infection.

Table S3: Findings at baseline (2012), 2015, and 2021 in intervention and control hospitals in Kyrgyzstan

	Intervention hospitals (%, 95% CI)						Control hospitals (%, 95% CI)					
**Study baseline (2012)**	**H1 N=120**	**H5 N=120**	**H6 N=123**	**H9 N=119**	**H10 N=111**	***Average****	**H2 N=112**	**H3 N=118**	**H4 N=118**	**H7 N=121**	**H8 N=120**	***Average*****
Unnecessary hospitalizations (%)	50	10	22.7	47.37	88.2	***43.7 (22.6-87.1)***	78.1	52.5	12.2	46.3	57.5	***49.3 (5.5-93.1)***
Unnecessarily prolonged hospitalizations	78.9	89.2	91.4	78.6	96	***86.8 (57.2-100)***	100	92.3	94.7	94.1	97.1	***95.6 (77.7-100)***
Use of pulse oximeter	00.0	00.0	00.0	00.0	00.0	***0.0***	00.0	00.0	00.0	00.0	00.0	***0.0***
Antimicrobial prescription during hospitalization												
Diarrhoea	85.2	100	95.45	100	100	***96.1 (79.2-100)***		94.1	100.0		100.0	***98.0 (82.3-100)***
Respiratory infection	100	100	86.4	94.7	100	***96.2 (79.5-100)***	96.9	100	100	100	100	***99.4 (92.5-100)***
Oral rehydrated salts[1]	25.9	81.3	59.1	63.2	100	***65.9 (24.3-100)***		64.7	90.0		85.0	***79.9 (34.6-100)***
Zinc[1]	0.0	0.0	0.0	0.0	0.0	***0.0***		0.0	0.0		0.0	***0.0***
Referrals	40.0	92.5	56.8	76.3	52.9	***63.7 (21.6-100)***	56.3	62.5	85.4	70.7	67.5	***68.5 (27.8-100)***
Theophylline[2]	76.9	50.0	45.5	42.1	63.6	***55.6 (12.1-99.2)***	43.8	65.2	90.5	46.3	40.0	***57.2 (13.8-100)***
Calcium gluconate[2]	38.5	0.0	0.0	5.7	0.0	***8.8 (0.0-33.7)***	18.8	4.4	4.8	2.4	10	***8.1(0.0-32.0)***
**2015**												
Unnecessary hospitalizations	15	10	12.2	47.5	23.1	***21.6 (0.0-57.6)***	15	44.7	50	26.8	46.2	***36.5 (0.0-78.7)***
Unnecessarily prolonged hospitalizations	55.6	47.2	48.6	69	50	***54.1 (10.4-97.8)***	69.2	96.4	89.5	90.2	22.2	***73.5 (34.8-100)***
Use of pulse oximeter	2.5	25.0	92.7	5.0	64.1	***37.9 (0.0-80.4)***	50.0	2.6	36.8	0.0	42.5	***26.4 (0.0-65.0)***
Antimicrobial prescription during hospitalization												
Diarrhoea	80.0	5.9	50.0	100		***59.0 (10.8-100)***	90.0	94.4	100	90.0	100	***94.9 (75.6-100)***
Respiratory infection	53.3	100	82.6	95.0	97.4	***85.7 (54.9-100)***	100	100	90.9	90.5	100	***96.3 (79.7-100)***
Oral rehydrated salts[1]	96.0	94.1	88.9	75.0		***88.5 (57.2-100)***	90.0	72.2	100.0	70.0	85.0	***83.4 (50.9-100)***
Zinc[1]	0.0	0.0	0.0	0.0		***0.0***	0.0	0.0	0.0	0.0	0.0	***0.0***
Referrals	85.0	50.0	61.0	85.0	61.5	***68.5 (27.8-100)***	55.0	81.2	94.6	80.5	52.5	***72.8 (33.7-100)***
Theophylline[2]	6.7	0.0	0.0	15.0	23.1	***9.0 (0.0-34.0)***	0.0	35.0	4.6	52.4	10.0	***20.4 (0.0-55.7)***
Calcium gluconate[2]	0.0	0.0	0.0	5.0	0.0	***1.0 (0.0-9.7)***	0.0	10.0	0.0	4.8	5.0	***4.0 (0.0-21.1)***
**2021**												
Unnecessary hospitalizations	27.5	2.5	7.9	56.1	26.3	***24.1 (0.0-61.5)***	30.8	40	71.8	46.2	65	***50.8 (6.9-94.6)***
Unnecessarily prolonged hospitalizations	50.0	48.7	11.4	72.0	41.2	***44.7(1.1-88.2)***	86.8	96.3	93.3	45	78.3	***79.9 (44.8-100)***
Use of pulse oximeter	45.0	100	94.7	34.2	71.1	***69.0(28.4-100)***	95.0	40.0	56.4	53.9	50.0	***59.0 (15.9-100)***
Antimicrobial prescription during hospitalization												
Diarrhoea	66.7	29.4	52.9	60.0	83.3	***58.5 (15.3-100)***	100	93.3	68.8	100	77.8	***88.0 (59.5-100)***
Respiratory infection	89.5	100	100	100	84.4	***94.8 (75.3-100)***	100	100	87.0	96.9	100	***96.8 (81.3-100)***
Oral rehydrated salts[1]	100	100	100	86.7	66.7	***90.7 (65.2-100)***	94.7	93.3	37.5	100	72.2	***79.5 (44.2-100)***
Zinc[1]	0.0	0.0	0.0	0.0	0.0	***0.0***	0.0	20.0	0.0	28.6	0.0	***9.7(0.0-35.7)***
Referrals	47.5	38.5	57.9	63.4	56.7	***52.8 (9.0-96.6)***	42.5	55	64.1	69.2	42.5	***54.7(11.0-98.3)***
Theophylline[2]	10.5	0.0	4.8	0.0	0.0	***3.1 (0.0-18.2)***	0.0	8.0	4.4	28.1	0.0	***8.1(0.0-32.0)***
Calcium gluconate[2]	0.0	0.0	0.0	0.0	0.0	***0.0***	0.0	0.0	0.0	0.0	0.0	***0.0***

95% CI: 95% Confidence Intervals; H: Hospital; *Cluster-level summary statistic for intervention hospitals; **Cluster-level summary statistic for control hospitals;1. Children with diarrhoea; 2. Children with respiratory infection.

Table S4: Findings at baseline (2012), 2015 and 2021 in intervention hospitals in Tajikistan

**Study baseline (2012)**	**Intervention hospitals (%, 95% CI)**								
	**H11 N=54**	**H12 N=47**	**H13 N=60**	**H14 N=74**	**H15 N=59**	**H16 N=95**	**H17 N=59**	**H18 N=113**	***Average****
Unnecessary hospitalizations (%)	61.5	16.7	80	35.0	45.0	55.6	50.0	46.7	***48.8 (14.2-83.5)***
Unnecessarily prolonged hospitalizations	80.0	90.0	100	92.3	100	100	80	83.3	***90.7 (70.6-100)***
Use of pulse oximeter	00.0	00.0	00.0	00.0	00.0	00.0	00.0	00.0	***0.0***
Antimicrobial prescription during hospitalization									
Diarrhoea			100	33.3	0.0	100	87.5		***86.7 (22.1-100)***
Respiratory infection	100	100	100	58.8	100	95.0	100	87.1	***92.6 (74.5-100)***
Oral rehydrated salts[1]			55.6	66.7	100	100	62.5		***77.0 (40.0-100)***
Zinc[1]			0.0	100	100	11.1	50.0		***52.2 (8.4-96.0)***
Referrals	7.1	0.0	20	15.0	100	93.1	91.7	22.6	***43.7 (9.3-78.1)***
Theophylline[2]	100.0	91.67	36.4	0.0	42.1	35.0	0.0	22.6	***41.0 (6.9-75.0)***
Calcium gluconate[2]	85.7	91.7	36.4	5.9	15.8	50.0	25.0	35.5	***43.3 (8.9-77.6)***
**2015**									
Unnecessary hospitalizations	46.7	46.7	35.0	52.4	21.1	40.7	66.7	50.0	***44.9 (10.4-79.4)***
Unnecessarily prolonged hospitalizations	62.5	100	30.8	70	66.7	75.0	40.0	53.8	***62.4 (28.8-95.9)***
Use of pulse oximeter	00.0	00.0	00.0	00.0	00.0	00.0	00.0	00.0	***0.0***
Antimicrobial prescription during hospitalization									
Diarrhoea	0.0	25.0	25.0	45.5	16.7	100	93.3	0.0	***41.0 (4.5-71.9)***
Respiratory infection	92.3	90.9	91.7	58.3	100	100	100	92.6	***90.7 (70.6-100)***
Oral rehydrated salts[1]	100	75.0	87.5	90.9	100	50	93.3	100	***87.1 (36.8-100)***
Zinc[1]	50.0	100	50.0	90.9	100	0.0	93.3	76.9	***70.1(38.4-100)***
Referrals	0.0	20.0	55.0	8.7	100	88.9	95.5	22.5	***48.8 (14.2-83.5)***
Theophylline[2]	61.5	9.1	0.0	0.0	15.4	8.0	14.3	7.4	***14.5 (0.0-38.8)***
Calcium gluconate[2]	15.4	0.0	0.0	8.3	0.0	4.0	0.0	0.0	***3.5 (0.0-16.1)***
**2021**									
Unnecessary hospitalizations	12.5	15.0	60.0	50.0	25.0	35.3	64.0	66.7	***41.1 (7.0-75.2)***
Unnecessarily prolonged hospitalizations	66.7	52.9	87.5	73.3	40.0	84.2	33.3	58.3	***62.0 (28.4-95.7)***
Use of pulse oximeter	8.0	00.0	00.0	3.2	00.0	7.7	00.0	16.7	***4.5 (0.0-18.7)***
Antimicrobial prescription during hospitalization									
Diarrhoea		50.0	100	35.7	27.3	100	100	27.3	***48.4 (27.1-98.7)***
Respiratory infection	100	100	100	94.1	100	100	100	100	***99.3 (93.3-100)***
Oral rehydrated salts[1]		100	100	85.7	90.9	0.0	100	90.9	***81.1 (52.1-100)***
Zinc[1]		100	100	100	90.9	0.0	93.8	100	***83.5 (56.1-100)***
Referrals	4.0	20	0.0	90.3	15.0	79.5	100	0.0	***38.6 (4.9-72.3)***
Theophylline[2]	68.0	0.0	21.4	11.8	0.0	5.4	11.1	16.1	***16.7 (0.0-42.6)***
Calcium gluconate[2]	64.0	0.0	0.0	11.8	0.0	13.5	11.1	0.0	***12.6 (0.0-35.5)***

CI: 95% Confidence Intervals; H: Hospital; *Cluster-level summary statistic for intervention hospitals; 1. Children with diarrhoea; 2. Children with respiratory infection.
Note: The number of medical records to review in each hospital for each year (2012, 2015 and 2021) was 40; this was chosen on the basis of previous similar work and the experience of the prior health systems evaluation (1,15). However, data collection in the first hospital allowed a realistic appraisal of time constraints, and the number of medical records to review for each subgroup was revised to 40 for 2021 and to 20 for each of the years 2012 and 2015.

Table S5: Findings in control hospitals in Tajikistan (2021)

2021	Control hospitals (%, 95% CI)										
	**H19 N=28**	**H20 N=28**	**H21 N=41**	**H22 N=40**	**H23 N=39**	**H24 N=35**	**H25 N=37**	**H26 N=34**	**H27 N=36**	**H28 N=34**	***Average****
Unnecessary hospitalizations	42.9	37.5	51.2	53.9	71.8	41.2	0.0	42.4	31.4	44.1	***41.6 (11.1-72.2)***
Unnecessarily prolonged hospitalizations	89.3	82.1	65.9	82.5	87.2	60.0	94.6	64.7	61.1	60.6	***74.8 (47.9-100)***
Use of pulse oximeter	0.0	0.0	0.0	0.0	7.7	0.0	48.7	0.0	2.8	0.0	***5.9 (0.0-20.5)***
Antimicrobial prescription during hospitalization											
Diarrhoea	100	66.7	90.5	100	100	100	93.3	90.9	100	100	***94.1 (79.6-100)***
Respiratory infection	100	94.7	100	100	100	100	100	100	97.1	89.5	***98.1 (89.7-100)***
Oral rehydrated salts[1]	66.7	22.2	100	0.0	90.0	22.2	66.7	27.3	0.0	66.7	***60.5(53.8-67.2)***
Zinc[1]	83.3	88.9	0.0	0.0	0.0	11.1	100	81.8	0.0	60.0	***42.5 (11.9-73.1)***
Referrals	78.6	50.0	41.5	47.5	35.9	28.6	46.0	100.0	27.8	88.24	***54.4 (23.5-85.3)***
Theophylline[2]	13.6	10.5	0.0	0.0	5.3	29.4	86.4	0.0	45.7	47.4	***23.8 (0.0 – 50.2)***
Calcium gluconate[2]	27.3	0.0	10.0	8.1	5.3	23.5	4.6	4.4	8.6	10.5	***10.2 (0.0 – 29.0)***

CI: 95% Confidence Intervals; H: Hospital; *Cluster-level summary statistic for control hospitals;1. Children with diarrhoea; 2. Children with respiratory infection.

Fig S1. Hospital selection and medical records selection in Kyrgyzstan and Tajikistan

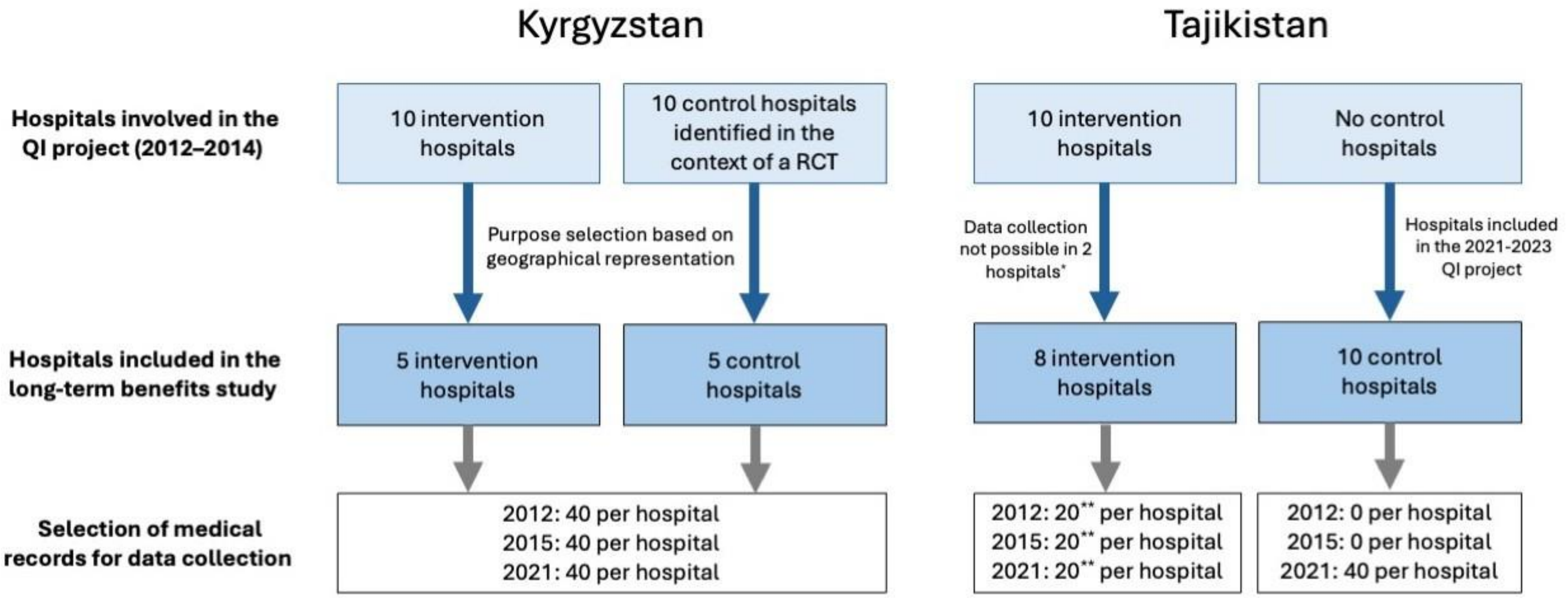

RCT: randomized controlled trial
*Visits to two hospitals were planned but could not be conducted due to security concerns at the time of data collection.
**The number of medical records reviewed was reduced to 20 because of logistical and time constraints.

Fig S2. Intervention and control hospitals in Kyrgyzstan

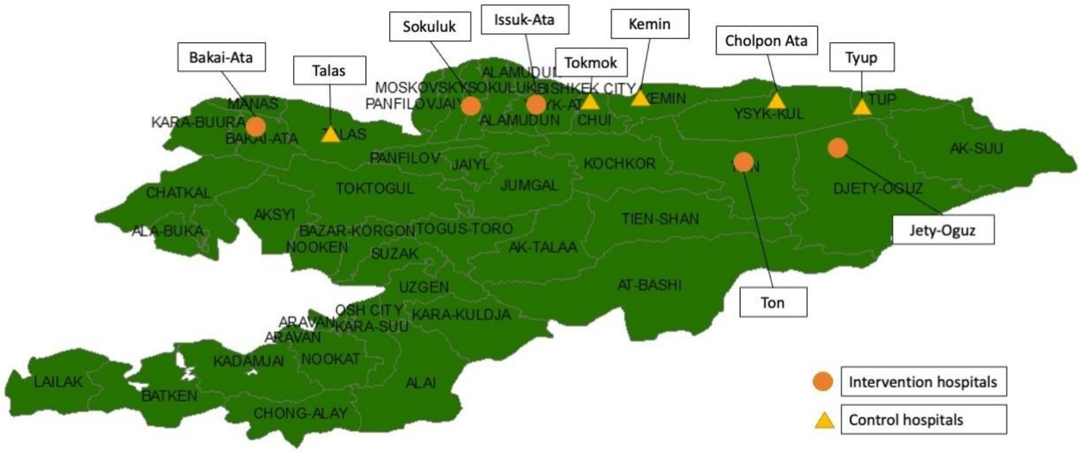

Fig S3. Intervention and control hospitals in Tajikistan

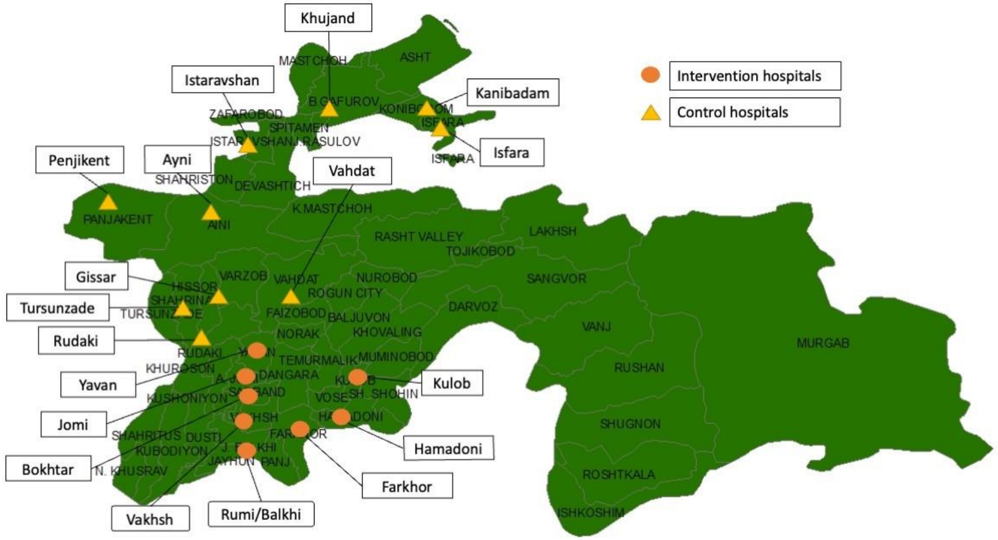

Supplement: Online Supplementary Document [file jogh-15-04133-s001.pdf]
